# Inpatient Falls and Orthopaedic Injuries in Elderly Patients: A Retrospective Cohort Analysis From a Falls Register

**DOI:** 10.7759/cureus.46976

**Published:** 2023-10-13

**Authors:** Mutaz AlSumadi, Masa AlAdwan, Amro AlSumadi, Chetan Sangani, Eugene Toh

**Affiliations:** 1 Trauma and Orthopaedics, Southport and Ormskirk Hospital NHS Trust, Southport, GBR; 2 Trauma and Orthopaedics, School of Medicine, University of Jordan, Amman, JOR

**Keywords:** trauma and orthopaedics, inpatient hip fracture, medical gerontology, injuries from falls, risk factors of falls, inpatient harm, inpatient falls

## Abstract

Background

Hospital inpatient falls have been a major area of concern in the healthcare setting. This poses a multifaceted challenge to healthcare systems, as elderly patients are at increased risk of harm and significant morbidity secondary to inpatient falls. In addition, hospital admission increases the risk of falls in acutely unwell elderly patients. There remains little consensus on best practices in reducing inpatient falls. With this, lies the risk to life or quality of life to this cohort of patients. Moreover, it is not evident whether orthopaedic injuries sustained by elderly patients in hospital and their management, including rehabilitation, has evolved with time.

Methodology

This was a retrospective cohort analysis of all inpatient falls over a three-year period in a single UK District General Hospital. A total of 101,183 acute admissions were analysed. All falls were identified and categorised into harm categories according to National Patient Safety Alerts. Patients sustaining moderate harm or more were assessed to determine injuries sustained, patient-associated factors, factors surrounding the fall, management incurred, length of stay, and financial burden incurred.

Results

A total of 101,183 admissions were analysed revealing a total of 2,453 in-patient falls. The rate of inpatient falls was 2.42%. Of these, 49 (1.98%) patients sustained moderate harm or more. Patient-related factors included age and comorbidities; 82% of patients were above the age of 75, and 78% of patients had three or more medical comorbidities. Fall-related factors leading to moderate harm or more included time of fall and ward. Most falls occurred out of hours (80%) and in acute medical wards (69%). The average length of stay following fall was 2.4 weeks per patient and a combined 110 weeks in the three-year period. In non-deceased patients, increased dependency and reduced mobility at discharge were noted. The total hospital annual financial burden due to moderate harm or more following an inpatient fall was approximately £123,490.00. Length of stay was the major contributor to this (£90,090.00 annually).

Conclusions

Inpatient falls remain a considerable patient safety issue, with orthopaedic injuries playing a central role in harm to patients following these falls. These also pose considerable service and financial costs to healthcare organisations. Further work is needed to identify best practices in in-hospital fall prevention and streamlining post-fall management and rehabilitation.

## Introduction

Hospital inpatient falls have been a major area of concern over the years in the healthcare setting due to their potential to precipitate head or orthopaedic injuries [1]. Although not all inpatient falls may be serious, some, especially orthopaedic injuries such as hip fractures, necessitate surgical intervention with consequent implications on length of stay and healthcare expenditure [2]. This is of particular importance in hospitalised patients, as inpatient falls are the most frequently reported safety incidents in the National Health Service (NHS) [3].

This poses a multifaceted challenge to healthcare systems, as elderly patients are at increased risk of fragility fractures and significant morbidity secondary to these [4]. In addition, hospital admission increases the risk of falls in acutely unwell elderly patients due to a variety of predisposing factors, such as an alien environment and associated medical comorbidities [5-7].

Despite advances in nursing and medical care for patients to reduce the risk of inpatient falls, the risk remains considerable, with varying incidence reported between 2% and 11% [8-13]. In the UK, inpatient falls have been reported at 2.7%, corresponding to 247,000 inpatient falls annually in England alone [14]. This is accompanied by an apparent increase in age-adjusted fall mortality rates in recent years and the increased complexity of elderly patients in hospitals [15].

These concerns have initiated increased focus on the prevention of inpatient falls and consequent fragility fractures with interventions and the development of guidelines by the National Institute for Health and Care Excellence (NICE), British Geriatric Society (BGS), and British Orthopaedic Association (BOA) [16].

There remains little consensus on best practices in reducing inpatient falls. With this lack of clarity lies the ongoing prevalence of life or quality of life-altering injuries for this cohort of patients [17,18]. Moreover, it is not evident whether orthopaedic injuries sustained by elderly patients in hospital and their management, including rehabilitation, has evolved with time.

The primary aim of this study was to assess orthopaedic injuries sustained by inpatients in a single UK district general hospital, as well as factors surrounding the falls leading to these. Secondary outcome objectives were assessing surgical management incurred, the effect on hospital stay, functional status and costs incurred, and comparing these trends to our previous study [2].

## Materials and methods

Study design and population

This was a retrospective cohort analysis from a single UK district general hospital of all patients above the age of 60 sustaining an inpatient fall over a three-year period. The hospital has 313 inpatient beds providing care to approximately 224,400 people across its catchment. Data were gathered from three consecutive years between June 2020 and June 2023.

Falls were defined according to the definition outlined by the World Health Organization as ‘an event which results in a person coming to rest inadvertently on the ground or floor or other lower level’.

Harm sustained secondary to falls was classified according to National Patient Safety Alerts (NPSA) guidance set out in the UK into no harm, low harm, moderate harm, severe harm, and death. Definitions are presented in Table 1 [19].

**Table 1:** Definitions of harm according to the NPSA. Categories of harm and their definitions sustained by patients following a patient-related safety incident (inpatient fall in this paper), as per the guidance set out by the NPSA. NPSA = National Patient Safety Agency

NPSA harm severity grading	Definition
No harm	No harm sustained
Low harm	Minimal harm - patient(s) required extra observation or minor treatment
Moderate harm	Short-term harm - patient(s) required further treatment or procedure
Severe harm	Permanent or long-term harm.
Death	Death (caused directly by the Patient Safety Incident)

Inclusion and exclusion criteria

Inclusion criteria included all patients above the age of 60 who had sustained a fall while an inpatient in an acute bed for all-cause admissions. Patients sustaining falls while attending outpatient appointments or attending the accident and emergency department were excluded.

Primary and secondary outcome objectives

The primary outcome objectives were to determine the rate of inpatient falls, classify these into harm categories, and identify injuries sustained by patients sustaining moderate harm or more.

Secondary outcome objectives were to examine factors surrounding falls leading to moderate harm or more, management incurred, trends in costs, and rehabilitation of orthopaedic injuries following inpatient falls in comparison to previous results from our unit.

Data and image collection and analysis

All patients who had sustained a fall were identified using the hospital’s incident reporting system DATIX™ (London, UK). Outcomes of these falls were recorded according to the definitions of harm by NPSA described above.

Inpatient falls leading to moderate harm or more were identified and included in a more detailed analysis. Data for these patients were collated from Electronic Patient Records (EPR), Picture Archiving and Communication System (PACS), Falls Audit Register, discharge summaries, and electronic care flow records (CARE Flow).

Data analysis

Electronic data collation and processing were performed using Microsoft Excel and Word (Microsoft Corp., Redmond, WA, USA). The collated data were organised using tables corresponding to the results obtained concerning the study variables. The analysis included patient factors such as sociodemographics, medical comorbidities, and mobility before admission and at discharge). Factors surrounding the fall included date of fall, time, and ward, and factors surrounding the harm sustained included injury, management incurred, length of stay, and cost analysis. Cost estimates were gathered via the hospital’s Clinical Audit and the Finance Departments.

## Results

During the study timeframe (June 2020 to June 2023), we identified a total of 2,453 inpatient falls corresponding to 2.42% of admissions (total = 101,183 admissions). Of these, 77.2% (1,896 patients) sustained no harm secondary to an inpatient fall, 20.7% (508 patients) sustained low harm, 1.54% (38 patients) sustained moderate harm, and 0.44% (11 patients) sustained serious harm or death.

We identified 49 patients who had sustained moderate harm or more. In four patients, insufficient clinical documentation was available to include in the analysis; therefore, these patients were excluded from further analysis.

Injuries sustained

Orthopaedic injuries accounted for the majority (82%, 37 patients) of injuries leading to moderate harm or more. Intracranial haemorrhage led to the remaining 18% (8 patients) of moderate harm or more (Table 2). We assessed mortality associated with inpatient falls up to three months following the inpatient fall. These were associated solely with neck of femur fractures (seven patients, 64%) or intracranial haemorrhage (four patients, 36%).

**Table 2:** Injuries sustained by patients in the cohort and associated mortality in the study group. Injuries sustained in patients who had sustained moderate harm or more secondary to an inpatient fall, represented in the number of patients and percentage, with corresponding mortality in the study group.

Injury sustained	Number of patients (%)	Death secondary to injury (%)
Neck of femur fracture	19 (42%)	7 (64%)
Intracranial haemorrhage	8 (18%)	4 (36%)
Distal radius fracture	4 (9%)	0 (0%)
Proximal humeral fracture	3 (7%)	0 (0%)
Pubic rami fracture	2 (4%)	0 (0%)
Clavicle fracture	2 (4%)	0 (0%)
Ankle fracture	1 (2%)	0 (0%)
Dislocation total hip replacement/Hip hemiarthroplasty	1 (2%)	0 (0%)
Vertebral fracture	1 (2%)	0 (0%)
Tibial fracture	1 (2%)	0 (0%)
Orbital fracture	1 (2%)	0 (0%)
Elbow fracture	1 (2%)	0 (0%)
Shoulder dislocation	1 (2%)	0 (0%)
Total	45	11

Patient demographics

Regarding patient-related factors and harm following an inpatient fall, we found advanced age (>75 years old) being associated with a majority of instances (82%, 37 patients) of inpatients sustaining moderate harm or more following an inpatient fall. Expectedly, patients admitted to the hospital were comorbid with underlying medical conditions, with 55.5% (25 patients) of patients sustaining moderate harm or more having four or more comorbidities. There were 25 (55.5%) females and 20 (44.4%) males in the cohort analysis (Table 3). Reflecting on mortality and patient-related factors, we found advanced age (>75 years old) to be associated with 91% of mortality (10 patients) following an inpatient fall, with one outlier (9%) at 60 years old with extensive underlying medical comorbidities.

**Table 3:** Demographics and associated comorbidities of patients in the study group. Patient-associated factors in inpatient falls leading to moderate harm or more, demonstrating age of patients, number of associated medical comorbidities, and gender of patients, represented in the number of patients and percentages.

	Number of patients (%)
Patient age sustaining moderate harm or more	
Age >85	21 (46.6%)
Age 75–85	16 (35.5%)
Age 60–74	8 (17.7%)
Number of comorbidities in patients sustaining moderate harm or more	
4 or more comorbidities	25 (55.5%)
3 comorbidities	10 (22.2%)
2 or less comorbidities	10 (22.2%)
Patient gender sustaining moderate harm or more	
Female	25 (55.5%)
Male	20 (44.4%)

We also examined patients’ methods of mobility at admission and at discharge. Examining these, we found trends of increased dependency and reduced mobility findings at discharge compared to pre-admission, as summarised in Table 4. At discharge, 11 (24.4%) patients were deceased and thus not included in the discharge mobility assessment.

**Table 4:** Mobility before admission and at discharge of the patients. Assessment of patient mobility of study group patients, categorised into independent mobility, with a mobility aid (stick or frame) or assistance of an individual with or without a mobility aid, assessed at admission and discharge. Represented in the number of patients and percentages.

Method of mobility	Pre-admission: number of patients (%)	At discharge: number of patients (%)
Independent	27 (60%)	6 (18%)
Walking stick	5 (11%)	1 (3%)
Walking frame	4 (9%)	5 (15%)
Assistance of one or more with or without a mobility aid	9 (20%)	22 (65%)

Fall demographics

When assessing fall-related factors in patients whose fall led to moderate harm or more, more falls occurred outside of normal working hours (36 patients, 80%), with equal numbers occurring between midnight and 8 AM (18 patients, 40 %) and any time after 4 PM to midnight (18 patients, 40%) (Table 5). The type of ward in which inpatient falls occurred showed a higher incidence in medical wards (31 patients, 69%), However otherwise appeared to be distributed cohesively between various types of wards, as summarised in Table 6.

**Table 5:** Timing of inpatient falls and completion of admission falls risk assessment. Timing of falls leading to moderate harm or more (categorised into eight-hour windows, normal working hours 8 AM to 4 PM, evening 4 PM to midnight, and nighttime midnight to 8 AM) and completion of the hospital’s falls risk assessment proforma at admission, represented in the number of patients and percentages.

Timing of fall incident	Number of falls (%)
00:00 to 07:59	18 (40%)
08:00 to 15:59	9 (20%)
16:00 to 23:59	18 (40%)
Completion of risk assessment at admission	Number of patients (%)
Risk assessment complete	7 (16%)
Risk assessment not complete	38 (84%)

**Table 6:** Place of injury. Distribution of falls leading to moderate harm or more according to the ward in which the fall occurred, represented in the number of falls and percentage.

Place of fall	Number of patients (%)
Gastroenterology	6 (14%)
A&E Inpatients	6 (14%)
Geriatric Ward	5 (11%)
Short Stay Unit	5 (11%)
Respiratory Ward	5 (11%)
Acute Medical Unit	4 (9%)
Cardiac Unit	4 (9%)
Rehabilitation Ward	4 (9%)
Stroke Ward	2 (4%)
Surgical Ward	2 (4%)
Spinal Unit	1 (2%)
ITU	1 (2%)

Length of stay and financial burden

The length of stay was recorded following inpatient falls and is summarised below. Length of stay following fall was categorised into less than one week, one to three weeks, and more than three weeks. Length of stay following an inpatient fall varied from less than one week (11 patients, 28%), one to three weeks (15 patients, 37%), and more than three weeks (14 patients, 35%).

The average additional length of stay was 2.4 weeks per patient following an inpatient fall leading to moderate harm or more, and the total combined additional length of stay was 110 weeks, as presented in Table 7.

**Table 7:** Length of stay following inpatient falls. Time from inpatient falls to discharge from hospital, with an average additional length of stay following inpatient fall per patient and total combined additional stay for all patients sustaining moderate harm or more following an inpatient fall, represented in the number of patients and percentage.

Length of stay following fall	Number of patients (%)
Less than 1 week	11 (28%)
1–3 weeks	15 (37%)
More than 3 weeks	14 (35%)
Total combined additional stay	110 weeks
Average additional stay per patient	2.4 weeks

Associated costs have been calculated and categorised into treatment-related costs and hospital admission costs. The daily cost of excess bed occupancy in the UK from NHS England was estimated at £351.00 (2016/2017 prices) and applied to the length of hospital stay to estimate the cost of hospital stay [20]. On average, in our patient sample, this equated to 16.8 days (2.4 weeks), costing £5896.80 in hospital stay following a fall leading to moderate harm or more per patient. In total, this led to a £270,270.00 financial burden in hospital stay costs following an inpatient fall in the three-year period or £90,090.00 annually.

The cost of treating hip fractures in the UK is calculated via remuneration, in which reimbursement is paid to hospitals following the management of conditions. Remuneration costs for hip fractures obtained via the National Hip Fracture Database have been utilised, which are £5,695.00 for a dynamic hip screw or an intramedullary nail surgical fixation of a hip fracture, and £6,392.00 for a hip hemiarthroplasty [21]. The cost of a plaster cast has been estimated at £300.00 through guidance from the finance department [2].

According to this, in our patient sample, we estimated a total financial burden of £95,999.00 in surgical management incurred secondary to inpatient falls in the three-year period or £32,000.00 annually. Treatment costs incurred for patients sustaining moderate harm or more following an inpatient fall are summarised in Table 8. Table 9 presents the management incurred for injuries sustained following inpatient falls.

**Table 8:** Estimated treatment costs for patients’ orthopaedic injuries in the hospital. Cost estimations based on figures from NHS England and the hospital’s finance department for an additional length of stay and management of injuries in patients following inpatient falls leading to moderate harm or more in our study group. N/A = not applicable

Category of treatment cost incurred	Total number (average cost per unit of category)	Total cost
Hospital admission per week	110 weeks (£2,457.00)	£270,270.00
Hip hemiarthroplasty	7 operations (£6,392.00)	£44,744.00
Hip surgical fixation	9 operations (£5,695.00)	£51,255.00
Plaster cast	14 casts (£300.00)	£4200.00
Total	N/A	£370,469.00

**Table 9:** Management incurred for injuries sustained following inpatient falls. Management necessitated for patients sustaining moderate harm or more following an inpatient fall, represented in the number of patients and percentages. DHS = dynamic hip screw; IM = intramedullary

Management incurred in managing patients with moderate harm or more	Number of patients (%)
Dynamic hip screw DHS surgical fixation	5 (11.%)
Intramedullary nail IM surgical fixation	4 (9%)
Hip hemiarthroplasty	7 (15%)
Plaster cast/Splint	14 (31%)
Observation/Rehabilitation	15 (34%)

## Discussion

There has been an increasing body of literature examining inpatient falls [8,9]. Certainly, inpatient falls inherently carry the risk of injuries and serious sequelae to patients and create pressure on healthcare services in terms of further management and cost incurred following these adverse events.

This increasing concern has been met with recently improved awareness and purchase into inpatient fall prevention. This is reflected in the National Audit of Inpatient Falls (NAIF) examining inpatient falls leading to neck of femur fractures [22]. NAIF also reports increased compliance with the audit in recent years. Despite this, most studies and national audits have either focused on fall prevention and risk assessments [10,12] or solely on neck femur fractures [22]. There is a paucity of data examining orthopaedic injuries sustained following inpatient falls. There is also limited data on the management of these and cost considerations for orthopaedic injuries sustained by inpatients.

Our results showed a 2.4% incidence of falls in inpatients, which is comparable to results in the literature [12]. In our sample, orthopaedic injuries were the main pathology accounting for the majority (82%) of inpatient falls leading to moderate harm or more. Undoubtedly, neck of femur fractures represented a significant portion of this (42%). However, there was a wide range of musculoskeletal injuries, summarised in Table 2, that we believe highlight the importance of focusing on examining orthopaedic injuries in inpatient falls.

Falls with moderate harm sequelae or more occurred more commonly with advanced age, with approximately half (47%) occurring in patients above 85 years old, suggesting the termed ‘oldest-old’ age group to be particularly at risk [23]. Mortality was also almost exclusively (91%) associated with advancing age (>75 years old) signifying the importance of advancing age in sustaining harm in in-hospital falls. This suggests that these patient groups should possibly be particularly identified and targeted.

Most patients in our group (78%) sustaining moderate harm or more did have three or more medical comorbidities, suggesting that particular interest in these patients in the context of harm following inpatient falls is possibly of value.

This is of particular importance in relation to NICE guidelines recommending against risk assessment tools in predicting falls in inpatients and recommends consideration of all patients above the age of 65 as ‘at risk’. This does pose the question of a more targeted approach, identifying patients at risk of more serious harm rather than a blanket consideration for all patients [24].

Orthopaedic injuries sustained carried a variety of management incursions, including hip hemiarthroplasty, surgical fixation of long bone fractures (sliding hip screw, intramedullary nail device, plates, and screws), and plaster casting. These combined with additional length of stay (Table 8) led to a notable pressure on hospital beds and financial burden in management costs. Compared to results published from our unit previously by Nadkarni et al., orthopaedic injuries have led to an apparent longer post-fall additional stay per patient (1.35 weeks vs. 2.4 weeks) [2]. This can be reflective of a more comprehensive, albeit burdensome, rehabilitation and discharge process. Nevertheless, this does add to hospital pressure and financial burden.

This study does have limitations. It is limited by the retrospective nature of the study design, with the possible effect of this on compounding factors in data interpretation. Despite this, however, prospective randomised controlled trials in this context are challenging and most evidence available is retrospective in nature, as revealed by LeLaurin et al. [12]. We do note that the study, despite a large patient population and timeframe, is possibly limited by the post-fall rehabilitation and discharge data which can be influenced by a variety of variables.

Our recommendation for future work would be to assess risk factors contributing to harm following an inpatient fall while minimising compounding factors, a suggested model would be a case-control study in a matched 1:3 ratio between inpatient fallers who have sustained moderate harm or more (case) and those who have fallen without harm (control).

## Conclusions

Falls in hospitals remain a considerable patient safety issue, with orthopaedic injuries playing a central role in harm to patients following inpatient falls, with apparent higher mortality compared to community falls, with features of increased dependence at discharge. Therefore, attention should be paid to inpatient orthopaedic injuries, for example, highlighting those at the highest risk of harm rather than simply those at risk of falls.

Inpatient falls also pose considerable service and financial costs to healthcare organizations, mainly highlighted in the length of stay following fall and surgical management costs. Further work is needed to identify best practices in in-hospital fall prevention and streamlining post-fall management and rehabilitation.

## References

[1] Najafpour Z, Godarzi Z, Arab M, Yaseri M (2019). Risk factors for falls in hospital in-patients: a prospective nested case control study. Int J Health Policy Manag.

[2] Nadkarni JB, Iyengar KP, Dussa C, Watwe S, Vishwanath K (2005). Orthopaedic injuries following falls by hospital in-patients. Gerontology.

[3] Morris R, O'Riordan S (2017). Prevention of falls in hospital. Clin Med (Lond).

[4] Currie L (2008). Fall and injury prevention. Patient Safety and Quality: An Evidence-Based Handbook for Nurses.

[5] Vu MQ, Weintraub N, Rubenstein LZ (2004). Falls in the nursing home: are they preventable?. J Am Med Dir Assoc.

[6] Vassallo M, Sharma JC, Allen SC (2002). Characteristics of single fallers and recurrent fallers among hospital in-patients. Gerontology.

[7] Chen X, Van Nguyen H, Shen Q, Chan DK (2011). Characteristics associated with recurrent falls among the elderly within aged-care wards in a tertiary hospital: the effect of cognitive impairment. Arch Gerontol Geriatr.

[8] Gillespie LD, Gillespie WJ, Robertson MC, Lamb SE, Cumming RG, Rowe BH (2003). Interventions for preventing falls in elderly people. Cochrane Database Syst Rev.

[9] Cuttler SJ, Barr-Walker J, Cuttler L (2017). Reducing medical-surgical inpatient falls and injuries with videos, icons and alarms. BMJ Open Qual.

[10] Heng H, Jazayeri D, Shaw L, Kiegaldie D, Hill AM, Morris ME (2020). Hospital falls prevention with patient education: a scoping review. BMC Geriatr.

[11] de Souza AB, Röhsig V, Maestri RN (2019). In hospital falls of a large hospital. BMC Res Notes.

[12] LeLaurin JH, Shorr RI (2019). Preventing falls in hospitalized patients: state of the science. Clin Geriatr Med.

[13] Bernet NS, Everink IH, Schols JM, Halfens RJ, Richter D, Hahn S (2022). Hospital performance comparison of inpatient fall rates; the impact of risk adjusting for patient-related factors: a multicentre cross-sectional survey. BMC Health Serv Res.

[14] (2023). National audit of inpatient falls 2022. https://www.data.gov.uk/dataset/320f3a10-f81b-410e-95b9-ed33bb702a62/national-audit-of-inpatient-falls-2022.

[15] Cuttler SJ, Barr-Walker J, Cuttler L (2017). Reducing medical-surgical inpatient falls and injuries with videos, icons and alarms. BMJ Open Qual.

[16] (2023). British Geriatric Society (BGS). 2018 NICE impact report on falls and fragility fractures. https://www.bgs.org.uk/resources/2018-nice-impact-report-on-falls-and-fragility-fractures.

[17] Montero-Odasso M, van der Velde N, Martin FC (2022). World guidelines for falls prevention and management for older adults: a global initiative. Age Ageing.

[18] Loganathan A, Ng CJ, Tan MP, Low WY (2015). Barriers faced by healthcare professionals when managing falls in older people in Kuala Lumpur, Malaysia: a qualitative study. BMJ Open.

[19] (2023). National Reporting and Learning System. Degree of harm FAQ. https://www.england.nhs.uk/wp-content/uploads/2019/10/NRLS_Degree_of_harm_FAQs_-_final_v1.1.pdf.

[20] Guest JF, Keating T, Gould D, Wigglesworth N (2020). Modelling the annual NHS costs and outcomes attributable to healthcare-associated infections in England. BMJ Open.

[21] (2023). The National Hip Fracture Database. https://www.nhfd.co.uk/20/hipfractureR.nsf/0/9b0274d92959f71a80257bfb0067ff23/.

[22] (2023). Royal College of Physicians. National Audit of Inpatient Falls annual report (2021 clinical and 2022 facilities audit data). and.

[23] Lee SB, Oh JH, Park JH, Choi SP, Wee JH (2018). Differences in youngest-old, middle-old, and oldest-old patients who visit the emergency department. Clin Exp Emerg Med.

[24] (2023). Falls in older people: assessing risk and prevention. Clinical guideline [CG161]. Clinical guideline [CG161], Published.

